# Widespread Public Misunderstanding of Pivotal Trials for COVID-19 Vaccines May Damage Public Confidence in All Vaccines

**DOI:** 10.3389/fpubh.2022.847658

**Published:** 2022-03-11

**Authors:** John Gibson

**Affiliations:** Department of Economics, University of Waikato, Hamilton, New Zealand

**Keywords:** COVID-19, vaccination, randomized control trial (RCT), vaccine hesitancy, public understanding

## Introduction

Vaccine hesitancy—the reluctance or refusal to vaccinate despite the availability of vaccines—was already considered a major threat to public health, even before development of novel COVID-19 vaccines (1). Rapid development and roll-out of COVID-19 vaccines, with more than nine billion doses in 2021 of vaccines first developed in 2020, many using novel technologies like messenger-RNA, has created further hesitancy (2). This hesitancy may reflect lack of public knowledge about the vaccines (3). In particular, there may be misunderstanding about testing the vaccines underwent. This misunderstanding may lead to unrealistic public expectations about what mass vaccination will achieve. Unmet expectations may undermine public confidence in vaccines, leading to further vaccine hesitancy in future.

It is therefore interesting to study what the public understand about trials for the COVID-19 vaccines, as a separate but related aspect of the overall vaccine hesitancy issue. This commentary provides some evidence on widespread public misunderstanding of the pivotal trials underpinning approval of COVID-19 vaccines. This misunderstanding may create overly-optimistic expectations about real-world vaccine performance. When these expectations are not met it may provide more fuel for growth of vaccine hesitancy. This unexpected outcome would be an unwanted by-product of the COVID-19 pandemic and it will take considerable skill from public health officials and politicians to reset public expectations in order to avoid this erosion of confidence in vaccines.

## What Public Health Experts Know

Public health researchers and other readers of medical journals will know that pivotal randomized control trials (RCTs) underpinning approval of COVID-19 vaccines did not set out to, and did not, test if the vaccines prevent transmission of the SARS-CoV-2 virus. Nor did the trials test if the vaccines reduce mortality risk. A *BMJ* review of seven phase III trials for COVID-19 vaccines from Moderna, Pfizer/BioNTech, AstraZeneca (separately for US and UK), Janssen, Sinopharm and Sinovac found endpoints in each case were just reduced risk of COVID-19 symptoms (4). The trials were not designed to see if the vaccines reduce risk of infection. Helpfully, the review quotes Tal Zaks, chief medical officer of Moderna, so claims about not testing for protection against infection nor testing for reduced mortality risk are straight from the horse's mouth:

“…Our trial will not demonstrate prevention of transmission…because in order to do that you have to swab people twice a week for very long periods and that becomes operationally untenable.”

“…Would I like to know that this prevents mortality? Sure, because I believe it does. I just don't think it is feasible within the timeframe [of the trial]—too many people would die waiting for the results before we ever knew that.” [Zaks, quoted in Doshi [(4): p. 3]].

Likewise, readers of the *Journal of the American Medical Association* may recall a claim by health bureaucrats Walensky, Walke and Fauci that “clinical trials have shown that the vaccines authorized for use in the US are highly effective against COVID-19 infection, severe illness and death” was deemed sufficiently inaccurate to warrant publishing a comment refuting this claim (5, 6). The basis of the refutation was that the primary endpoint for the RCTs was symptoms of COVID-19; a less exacting standard than testing to show efficacy against infection, severe illness, and death.

## What the Public Know

The general public rarely read the *BMJ, JAMA* or other medical journals and instead their understanding of the COVID-19 vaccines, and the criteria they were trialed against, comes from statements made by politicians and health bureaucrats. These statements are often buttressed by health advertising and subtle public relations campaigns that co-opt seemingly independent commentators (7). It is therefore of interest to measure what the general public understand of the COVID-19 vaccine trials, to see if their understanding matches the reality.

To obtain evidence on public understanding of the pivotal vaccine trials, I added a question to a regularly fielded omnibus national poll that uses a sample of landlines and mobile phones to create representative estimates for the voting-age population of New Zealand. This country started COVID-19 vaccination slowly, initially relying on elimination to “keep it out,” but scaled up quickly over the 4 months prior to the poll. For example, on August 10, 2021 New Zealand ranked last in the OECD in vaccine doses per 100 people (at 44-per-100) but by early December, when the poll was fielded, it had risen 17 places in the OECD ranking with a vaccination rate of 153 doses per 100 people (8).

New Zealanders also have high public confidence in government. The share answering “Yes” to the question: “do you have confidence in the national government?” is 12 percentage points above the OECD average (9). Perhaps that is why there was little public disquiet when the Prime Minister claimed that in matters of COVID-19 and vaccines: “Dismiss anything else, we will continue to be your single source of truth” (10).

While the poll was in the field New Zealand's Parliament voted in vaccine passports that curtailed rights of the unvaccinated. Just 2 weeks earlier, thousands of education and health workers lost jobs due to vaccine mandates. The government had also just procured a small batch of AstraZeneca for people resisting the Pfizer vaccine used exclusively until then. Thus, aspects of what the vaccines were designed to do and how they had been tested should have been very salient for the public at the time.

The poll question was:

The vaccine for COVID-19 marketed by Pfizer is the main COVID vaccine available in New Zealand. Based on your own understanding, were the trials that allowed the authorization of this vaccine designed to:

a) Test if the vaccine prevents infection and transmission of SARS-CoV-2 (the virus that causes COVID-19)?
b) Test if the vaccine reduces the likelihood of getting symptoms of COVID-19?
c) Test if the vaccine reduces the likelihood of getting seriously sick and dying?
d) All of the above?

**Figure 1:**
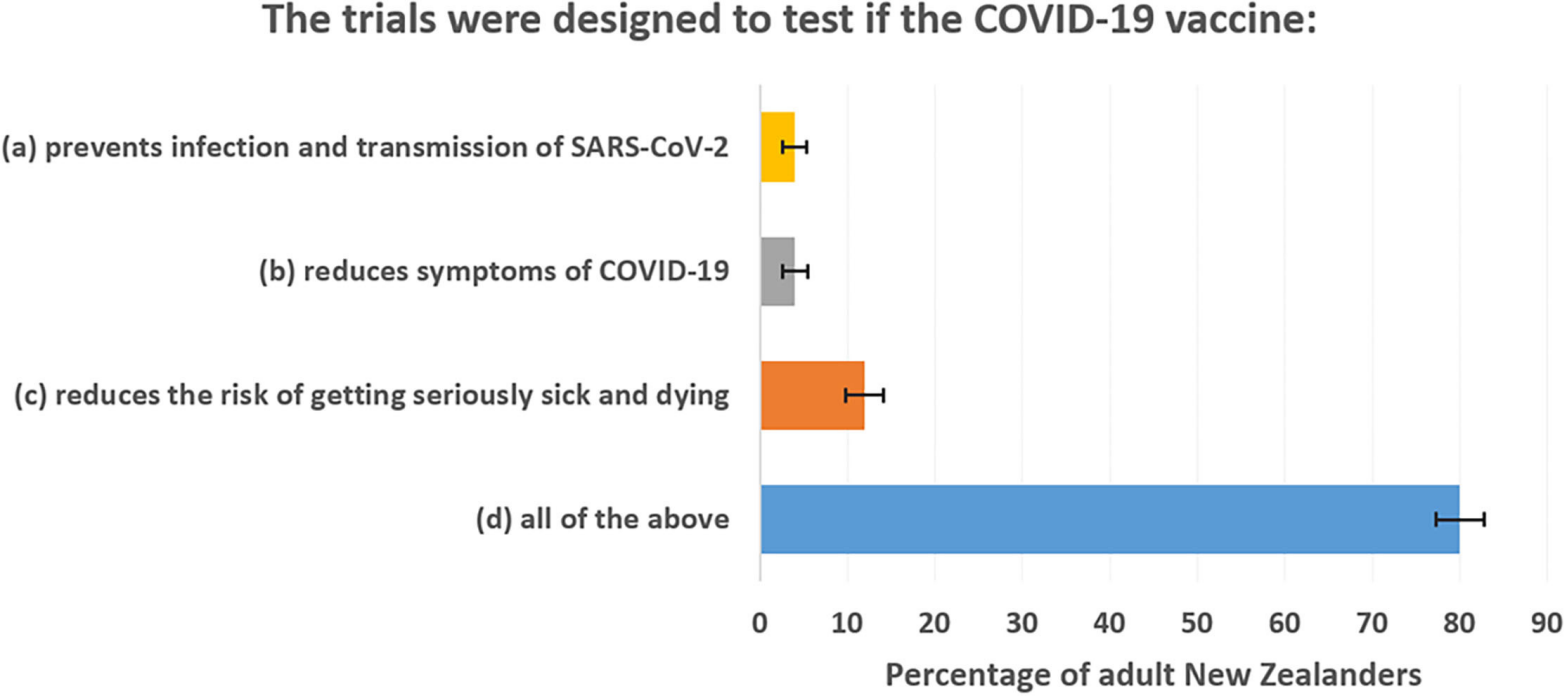
Public misunderstanding of what pivotal trials of COVID-19 vaccines were designed to show.

The correct answer to the survey question is option (b). Trials only tested for reduced risk of getting symptoms of COVID-19. Yet there were very high levels of misunderstanding with 96% of the adult population believing the trials also tested for protection against infection and/or for lowering mortality risk. Figure 1 shows percentage responses for each answer. The polling company (Curia Market Research) weighted the *n* = 852 responses to represent the overall voting-age adult population. The error bars show 95% confidence intervals. Based on these results, it seems that most of the public believe that the vaccines were trialed against more exacting criteria than is actually the case.

Before considering implications of this finding, a discussion of why respondents may view options (a), (b), and (c) distinct rather than as linked outcomes is necessary. Three features of SARS-CoV-2 and COVID-19 are relevant. First, asymptomatic spread implies that infection [option (a)] is not the same as symptomatic disease [option (b)]. In some studies, one-fifth to one-third of infections are from asymptomatic index cases (11, 12). Asymptomatic spread was also one reason given for lockdowns, as everyone, whether apparently healthy or not, was considered a risk of being an index case. This is likely to help respondents see options (a) and (b) as distinct.

For distinctions between options (b) and (c), age-specificity of COVID-19 mortality risk and the different clinical stages of the disease matter. Regarding age, compared to the Infection-Fatality Rate (IFR) for people age 0–34, the IFR for the 65–74 age group is 625 times higher, for 75–84 it is 2,125 times higher, and for 85+ it is 7,075 times higher (13). Someone with symptoms may have risk of death varying vastly with age so a vaccine that reduces one risk may not reduce the other proportionately, especially if efficacy varies with recipient age. Likewise, the various clinical stages of COVID-19, as it progresses from a viral infection with flu-like symptoms to viral pneumonia and pulmonary inflammation and then to pulmonary fibrosis (14), may delink risk of symptoms and risk of death. For example, a vaccine might stimulate an immune response to prevent progression into the pulmonary fibrosis stage, which would reduce mortality risk even if risk of flu-like symptoms was not reduced.

## Implications of what the Public don't Know

There are two, quite opposite, responses to finding the public believe COVID-19 vaccines were trialed against more exacting criteria than is actually the case. The first response is to argue that it is all semantics and what difference does it make, really, if vaccines were just trialed against reducing risk of COVID-19 symptoms. Surely, to do that they must also reduce risk of SARS-CoV-2 infection? However, that is just an assumption, not something tested in the trials. Moreover, one can assume the opposite; population-level infection risk may rise if a vaccine just reduces symptoms. Economists use the term “Peltzman effects” for risk compensation behavior, such as seat-belted drivers feeling more protected so driving less carefully and endangering cyclists and pedestrians (15). In the current context, vaccinees who think they are protected against infection may relax their use of other precautions. Infected people whose symptoms are suppressed by a vaccine might go out and spread the virus when otherwise they would have felt sick and stayed home.

The second response is to worry about loss of confidence in vaccines. The public believe the COVID-19 vaccines were trialed against more exacting standards than they truly were, so as real-world evidence mounts of vaccines not meeting these standards (e.g., in not providing durable protection against SARS-CoV-2 infection) some skepticism about the vaccines may arise. The possibility of this was foreshadowed by the Editor-in-Chief of the *BMJ*, about four months prior to the rollout of the COVID-19 vaccines:

“…we are heading for vaccines that reduce severity of illness rather than protect against infection, provide only short lived immunity, and will at best have been trialed by the manufacturer against placebo… damaging public confidence and wasting global resources by distributing a poorly effective vaccine…” (16).

It is important to maintain public trust in vaccine efficacy and safety in order to sustain gains made by vaccination programs (17). If too much is expected of COVID-19 vaccines, from a public believing they were trialed against more exacting standards than is actually so, as real-world performance fails to meet these exaggerated expectations there may be growing doubts about efficacy not just of these vaccines but of all vaccines. To avert the possible rise in vaccine hesitancy that may result from a mismatch between expectations and vaccine performance, a more explicit discussion of the criteria the COVID-19 vaccines were trialed against would be helpful. For example, if the public better understood that these vaccines offer protection against symptoms rather than against infection they could see them as part of a portfolio of measures for dealing with COVID-19. Other parts of that portfolio might include strengthening immune systems through improved diet and exercise, and for some groups perhaps supplementation with Vitamin D (18).

Some responsibility for this situation lies with politicians who have not been very frank with the public about limitations of the available COVID-19 vaccines. These same politicians have claimed to be the single source of truth, so this is a responsibility they have brought on themselves. For example, the New Zealand Prime Minister suggested that once vaccination rates are high enough, COVID-19 could be treated like measles (19). This was an unhelpful equivalence. Vaccination gives durable and almost complete (vaccine efficacy ≥97%) immunity against measles infection but it is now clear that the same cannot be expected for COVID-19.

## Conclusion

An unexpected cost of the COVID-19 pandemic may be erosion of public confidence in all vaccines. Paradoxically, some of this is from inflated claims made by pro-vaccine politicians and public health bureaucrats. These inflated claims contribute to misunderstanding by the public about the pivotal vaccine trials, creating unrealistic expectations about what the vaccines can do. It will be a difficult task to reset public expectations so that confidence in other vaccination programmes is not harmed. Even at this late stage, after nine billion doses have been administered, a full and frank discussion with the public about what the vaccines were designed to do, and the criteria they were tested against, is long overdue.

## Author Contributions

The author confirms being the sole contributor of this work and has approved it for publication.

## Conflict of Interest

The author declares that the research was conducted in the absence of any commercial or financial relationships that could be construed as a potential conflict of interest.

## Publisher's Note

All claims expressed in this article are solely those of the authors and do not necessarily represent those of their affiliated organizations, or those of the publisher, the editors and the reviewers. Any product that may be evaluated in this article, or claim that may be made by its manufacturer, is not guaranteed or endorsed by the publisher.
